# Ciphertext-Only Attack on Grayscale-Based EtC Image Encryption via Component Separation and Regularized Single-Channel Compatibility

**DOI:** 10.3390/jimaging12020065

**Published:** 2026-02-05

**Authors:** Ruifeng Li, Masaaki Fujiyoshi

**Affiliations:** Department of Computer Science, Tokyo Metropolitan University, 6-6 Asahigaoka, Hino 191-0065, Tokyo, Japan; li-ruifeng1@ed.tmu.ac.jp

**Keywords:** Encryption-then-Compression (EtC), grayscale-based image encryption, ciphertext-only attack, jigsaw puzzle solver, luminance component separation, regularized Mahalanobis compatibility, security evaluation

## Abstract

Grayscale-based Encryption-then-Compression (EtC) systems transform RGB images into the YCbCr color space, concatenate the components into a single grayscale image, and apply block permutation, block rotation/flipping, and block-wise negative–positive inversion. Because this pipeline separates color components and disrupts inter-channel statistics, existing extended jigsaw puzzle solvers (JPSs) have been regarded as ineffective, and grayscale-based EtC systems have been considered resistant to ciphertext-only visual reconstruction. In this paper, we present a practical ciphertext-only attack against grayscale-based EtC. The proposed attack introduces three key components: (i) Texture-Based Component Classification (TBCC) to distinguish luminance (Y) and chrominance (Cb/Cr) blocks and focus reconstruction on structure-rich regions; (ii) Regularized Single-Channel Edge Compatibility (R-SCEC), which applies Tikhonov regularization to a single-channel variant of the Mahalanobis Gradient Compatibility (MGC) measure to alleviate covariance rank-deficiency while maintaining robustness under inversion and geometric transforms; and (iii) Adaptive Pruning based on the TBCC-reduced search space that skips redundant boundary matching computations to further improve reconstruction efficiency. Experiments show that, in settings where existing extended JPS solvers fail, our method can still recover visually recognizable semantic content, revealing a potential vulnerability in grayscale-based EtC and calling for a re-evaluation of its security.

## 1. Introduction

With the rapid growth of cloud computing, social networking services, and AI-driven multimedia platforms, the secure transmission of digital images has become increasingly important. Encryption-then-Compression (EtC) systems [1,2,3,4,5] have emerged as a practical framework for enabling both efficient compression and privacy protection, as they preserve compatibility with JPEG [6] and support additional utilities such as reversible data hiding (RDH) [7,8,9,10,11]. Among various EtC designs, block scrambling–based perceptual encryption [3] has become one of the most widely adopted approaches due to its simplicity, format compliance, and low computational overhead, whereas recent alternatives like Joint Encryption and Compression (JEC) [12] and Encryption–Compression–Encryption (ECE) [13] have been proposed to balance compression with encryption or enhance security through double encryption. These approaches focus primarily on encryption design, while the security of widely deployed EtC systems against ciphertext-only reconstruction attacks remains an open problem.

A key security concern in block-scrambling–based EtC systems is that recovering an encrypted image can be formulated as assembling a large jigsaw puzzle. Over the past decade, jigsaw puzzle solver (JPS) algorithms—ranging from probabilistic and optimization-based approaches [14,15,16,17,18,19,20] to deep learning–based models [21,22]—have achieved remarkable success in reconstructing puzzles composed of thousands of pieces. As a result, several studies have shown that RGB-based EtC systems are vulnerable to visual reconstruction attacks based on JPS techniques [23,24]. In block-scrambling perceptual encryption, the encrypted image itself is directly observable and serves as the ciphertext, while the spatial layout and visual structure of the original image constitute essential plaintext information. Thus, reconstructing the arrangement of encrypted blocks can be regarded as a ciphertext-only attack (COA) in the EtC setting, even though it differs from COA in traditional cryptography.

To strengthen security against such reconstruction attempts in the baseline RGB block-scrambling EtC [3], Sirichotedumrong and Kiya [4,5] introduced a grayscale-based EtC system that serializes Y, Cb, and Cr into a pseudo-grayscale composite image and applies block-wise visual encryption. Compared with RGB-based pipelines that preserve cross-channel coupling at block boundaries, component serialization separates Y/Cb/Cr into distinct spatial regions, and per-block negative–positive inversion further disrupts boundary-consistency cues exploited by JPS-style solvers.

Previous work has evaluated this system using extended JPS attacks [23], which incorporate orientation handling and inversion compensation. Although these extended solvers are effective for RGB-based EtC systems, they still rely on consistent inter-channel correlations and boundary continuity. Once the color components are separated and luminance inversion is applied independently to each block, these assumptions break down, and extended JPS methods become fundamentally ineffective for grayscale-based EtC.

Despite rapid progress in generative and transformer-based solvers [25,26], they are not directly applicable to grayscale-based EtC images because channel serialization and per-block inversion break the boundary consistency they rely on. We show that this limitation is methodological: structural cues remain concentrated in the luminance (Y) component and can be exploited for reconstruction. The proposed framework introduces three key components: (i) Texture-Based Component Classification (TBCC) method that accurately separates encrypted blocks into luminance and chrominance groups; (ii) Regularized Single-Channel Edge Compatibility (R-SCEC) metric that adapts the Mahalanobis Gradient Compatibility (MGC) measure to single-channel gradients via Tikhonov regularization, enabling robust matching under rotation, flipping, and brightness inversion without relying on RGB inter-channel covariance; and (iii) Adaptive Pruning mechanism that eliminates redundant similarity evaluations among blocks with ambiguous edge structures.

Extensive experiments demonstrate that the proposed attack can successfully reconstruct visually meaningful images from the grayscale-based EtC system, whereas conventional extended JPS solvers fail completely. These findings constitute the first evidence that grayscale-based EtC image encryption is vulnerable to a carefully designed COA. Our results highlight the urgent need to re-assess the security assumptions of current EtC system designs and motivate the development of improved encryption strategies for future privacy-preserving multimedia applications.

## 2. Related Work

### 2.1. Compressible/Perceptual Encryption and EtC Architectures

Most existing image compression standards, such as JPEG, are optimized for the statistical properties of natural images. However, encrypted images typically exhibit noise-like distributions that disrupt these properties, making direct compression inefficient. For this reason, traditional Compression-then-Encryption (CtE) systems [27,28] have been widely used, in which images are first compressed in the clear and then encrypted before transmission.

Despite their compression efficiency, CtE systems require the exposure of unencrypted raw data during the compression stage. To address this privacy risk, EtC systems [1,2,3,4,5] have been proposed. In an EtC framework, content owners encrypt images on their own devices and then outsource only the encrypted data to third-party providers (e.g., cloud storage or social networking services), which subsequently perform compression. This architecture ensures that service providers never see the original image content, while still allowing the use of standard-compliant codecs such as JPEG [6] and supporting additional utilities such as reversible data hiding (RDH) [7,8,9,10,11].

Related approaches such as JEC [12] and ECE [13] focus on encryption system design and compression efficiency, whereas this study exclusively evaluates the security of block-scrambling-based EtC systems against ciphertext-only reconstruction attacks.

In parallel to EtC, compressible perceptual encryption (CPE) has been actively studied to enable privacy-preserving usage of images while maintaining codec compatibility. Imaizumi and Kiya [29] proposed a block-permutation-based scheme with independent processing of the RGB components, illustrating a practical design choice that balances secrecy and compressibility. More recently, Ahmad and Shin introduced IIB–CPE [30], which combines inter- and intra-block processing and further divides each block into sub-blocks to increase structural complexity under a secret key. Uzzal et al. [31] studied perceptual encryption for secure content-based image retrieval (SCBIR-PE), demonstrating encrypted-domain utility for retrieval tasks. Kiya et al. [32] provided an overview of compressible and learnable image transformations with a secret key and their applications, and Ahmad et al. [33] presented a comprehensive analysis of CPE from both compression and encryption perspectives. While these CPE/learnable-transformation studies emphasize utility preservation (e.g., inference or retrieval) under privacy constraints, our focus is orthogonal: we assess visual reconstruction risk under ciphertext-only attacks for block-scrambling-based EtC, particularly the grayscale-based architecture in [4,5], and we do not claim direct applicability to the above CPE frameworks. In addition to these CPE/learnable-transformation studies, EtC-compatible block-scrambling transforms have also been adopted for privacy-preserving image analysis on encrypted images (e.g., classification) in later works such as [34,35], further demonstrating the practical relevance of codec-compatible secret-key transforms beyond encryption design itself.

### 2.2. Jigsaw Puzzle Solvers and Visual Reconstruction Attacks

The objective of the jigsaw puzzle problem is to reconstruct the original spatial arrangements from a set of unordered fragments. The domain is broadly categorized into shape-based and content-based solvers. In the context of EtC systems, the focus is exclusively on square jigsaw puzzles (SJPs), where solvers must rely solely on visual content continuity.

From an algorithmic perspective, SJP solvers have evolved from traditional optimization strategies to modern deep learning-based approaches. Traditional solvers treat reconstruction as a combinatorial optimization problem, generally falling into three strategies: local search (greedy) algorithms like Gallagher [16] and Son et al. [17]; global search algorithms like the genetic algorithm by Sholomon et al. [18]; and hybrid approaches [19]. Notably, Gallagher [16] established a benchmark for handling puzzles with unknown rotation using Mahalanobis Gradient Compatibility (MGC). With the advent of deep learning, solvers based on Convolutional Neural Networks (CNNs) [21] and Generative Adversarial Networks (GANs) [22] have emerged. These methods excel at extracting semantic features to solve puzzles, even with eroded boundaries.

Comparative evaluations on small-scale puzzles (e.g., 3×3) indicate that deep learning-based solvers can achieve superior reassembly accuracy with computational overhead comparable to classic greedy methods [22]. However, these performance gains are typically demonstrated on puzzles with very limited piece counts, and their efficacy has not yet been established for the large-scale scenarios required in EtC cryptanalysis. In practical EtC settings, the number of blocks is significantly larger (e.g., 1024 blocks for a 256×256 image with a block size of 8×8 pixels). The resulting increase in combinatorial complexity remains a significant hurdle for current learning-based models, making optimization-based solvers a more robust baseline for large-scale ciphertext-only reconstruction in block-scrambling EtC environments.

In the specific domain of EtC cryptanalysis, Chuman et al. [23] proposed the Extended Jigsaw Puzzle Solver (EJPS), which adapts Gallagher’s local greedy search strategy [16] to attack RGB-based EtC systems [3]. As summarized in Table 1, while EJPS significantly extends the capabilities of traditional solvers by supporting block flipping and negative–positive transformations, it is primarily tailored for RGB-based systems that utilize color-channel correlations. To the best of our knowledge, the reconstruction of grayscale-based EtC architectures [4,5] remains a significant challenge due to the absence of such inter-channel constraints. In this paper, we provide a framework that specifically addresses single-channel encrypted images and covers a broader range of transformations than prior optimization-based methods (see the last row of Table 1).

## 3. Preliminaries

This section defines the EtC system models and the attack formulation used throughout the paper.

### 3.1. Encryption-Then-Compression (EtC) Systems

#### 3.1.1. RGB-Based EtC System

**Figure 1:**
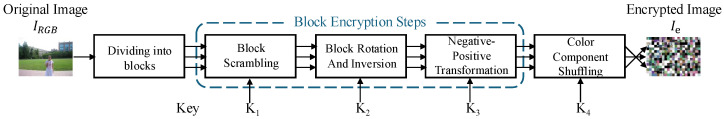
Schematic diagram of the block scrambling-based image encryption system for RGB images.

The conventional block-scrambling-based image encryption system [3] operates directly in the RGB color space. As illustrated in Figure 1, an image $I_{\mathrm{RGB}}$ with dimensions X×Y is first divided into non-overlapping blocks of size $B_{x}\times B_{y}$. Subsequently, a sequence of four block scrambling operations is applied to these blocks to generate the encrypted image $I_{e}$. The detailed encryption procedure is defined as follows:

1. Divide each color component of the RGB image $I_{\mathrm{RGB}}$ into blocks of $B_{x}\times B_{y}$ pixels.
2. Randomly permute the positions of these blocks using a pseudo-random number generator (PRNG) with a secret key $K_{1}$.
3. Apply random rotation and flipping to each block using another PRNG with a key $K_{2}$.
4. Invert the brightness of each block randomly using a third PRNG with a key $K_{3}$.
5. Shuffle the three color components within each block using a fourth PRNG with a key $K_{4}$.
6. Generate the final encrypted image $I_{e}$ by integrating the transformed block images.

#### 3.1.2. Grayscale-Based EtC System

**Figure 2:**
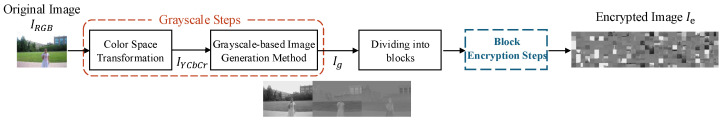
Schematic diagram of the grayscale-based block scrambling image encryption system. The system integrates color space conversion, horizontal concatenation, and three-layer block encryption.

In this paper, the target EtC system follows the grayscale-based framework described in recent literature [4,5]. In this framework, an RGB image is converted to the YCbCr color space, horizontally concatenated into a single grayscale image, and encrypted using three block-based operations: block permutation, block rotation/flipping, and pixel-wise inversion. These operations significantly disrupt spatial correlations and block ordering. Notably, compared to the conventional RGB-based scheme [3], this architecture was specifically designed to mitigate the reconstruction threats posed by extended jigsaw puzzle solver attacks, such as those demonstrated in [23]. The overall schematic of this system is illustrated in Figure 2, and the detailed encryption procedure is summarized below.

**Figure 3:**
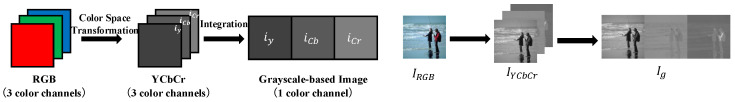
Illustration of grayscale-based image generation [4,5]. The RGB components are converted to YCbCr and concatenated horizontally to form $I_{g}$ (4:4:4) prior to encryption.

The specific encryption procedure consists of the following steps:

Step 1. Convert the RGB color image $I_{\mathrm{RGB}}$ of size X×Y to the YCbCr color space image $I_{\mathrm{YCbCr}}$, consisting of three channels: luminance ($I_{Y}$) and chrominance ($I_{\mathrm{Cb}}$, $I_{\mathrm{Cr}}$).
Step 2. Horizontally concatenate the three color channels ($I_{Y},I_{\mathrm{Cb}},I_{\mathrm{Cr}}$) to create a single grayscale image $I_{g}$ of size 3X×Y, as illustrated in Figure 3. Note that in this study, $I_{g}$ is generated without sub-sampling (4:4:4 format) to preserve maximum visual information for recovery analysis.
Step 3. Divide the concatenated grayscale image $I_{g}$ into non-overlapping blocks, each with dimensions $B_{x}\times B_{y}$.
Step 4. Block Encryption Operations: Apply the three operations as in Steps 2–4 of the RGB-based system described in Section 3.1.1.
Step 5. The result is the encrypted grayscale image $I_{e}$ of size 3X×Y, which is subsequently encoded by a standard JPEG compressor.

### 3.2. Jigsaw Puzzle Solver Attacks

#### Extended Jigsaw Puzzle Solver for EtC Systems

In the specific domain of EtC cryptanalysis, Chuman et al. [23] proposed the Extended Jigsaw Puzzle Solver (EJPS), which adapts Gallagher’s local greedy search strategy to attack RGB-based EtC systems.

**Figure 4:**
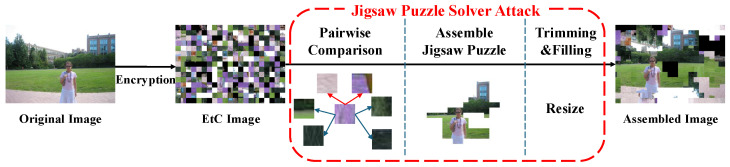
The complete framework of the Extended Jigsaw Puzzle Solver attack [23]. It consists of pairwise compatibility calculation using MGC, greedy puzzle assembly based on the calculated scores, and post-processing steps (trimming and filling).

The core contribution of this method is the introduction of a brute-force transformation search to handle encrypted blocks. As illustrated in Figure 4, the system first calculates compatibilities between all block pairs.

Let $B_{i}$ be a target block and $B_{j}$ be a candidate neighbor. The solver defines a set of composite transformation function $f_{p}$ to model the encryption operations:

(1) $$f_{p,k}\left(B_{j}\right)=f_{o}\circ f_{g}\circ f_{t}\circ f_{s}\left(B_{j}\right),$$

where the index *k* denotes a specific combination of transformation parameters, and the individual transformation components defined in [23] are as follows:

(2) $$\begin{array}{cc} \mathrm{Rotation}:\; & f_{s}\left(B_{j}\right),\;s\in \left\{0^{\circ },90^{\circ },180^{\circ },270^{\circ }\right\}, \end{array}$$

(3) $$\begin{array}{cc} \mathrm{Flipping}:\; & f_{t}\left(B_{j}\right),\;t\in \left\{0,H,V\right\}, \end{array}$$

(4) $$\begin{array}{cc} \mathrm{Inversion}:\; & f_{g}\left(B_{j}\right),\;g\in \left\{0,N\right\}, \end{array}$$

(5) $$\begin{array}{cc} \mathrm{Color}\;\mathrm{Shuffling}:\; & f_{o}\left(B_{j}\right),\;o\in \left\{0,\mathrm{GRB},\mathrm{RBG},\ldots \right\}. \end{array}$$

**Figure 5:**
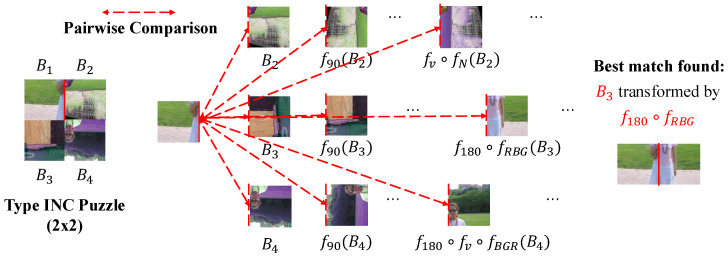
Illustration of the brute-force transformation search strategy employed by Chuman’s solver. For a target piece, the solver evaluates all transformed versions (rotation, flipping, inversion, and color shuffling) of candidate pieces to minimize the MGC distance.

To find the correct neighbor, the solver minimizes the compatibility score over all possible transformation combinations *k*. In other words, the solver enumerates all possible combinations and evaluates the corresponding composite transformation for each *k*, as shown in Figure 5. The final compatibility $C_{\mathrm{final}}$ is determined by the following:

(6) $$C_{\mathrm{final}}\left(B_{i},B_{j}\right)=\min_{k}\left(D_{\mathrm{MGC}}\left(B_{i},f_{p,k}\left(B_{j}\right)\right)\right),$$

where the MGC [16] is given by the following:

(7) $$D_{\mathrm{MGC}}\left(B_{i},B_{j}\right)=\sum_{n}{\left(g_{n}-\mu \right)}^{⊤}S^{-1}\left(g_{n}-\mu \right),$$

with $g_{n}$ denoting the RGB gradient vector at a boundary pixel, μ its mean, and *S* the 3×3 covariance matrix of boundary gradients in block $B_{i}$.

Chuman et al. [23] further classified puzzle types according to the active encryption operations and analyzed their search-space complexity. Table 2 summarizes the number of possible transformations per block for several representative types. For the RGB-based EtC system [3], all four operations (rotation, flipping, negative–positive inversion, and color shuffling) are active (Type INC), leading to |T|=8×2×6=96 candidate transformations per block.

For the target grayscale-based EtC system [4,5], two additional challenges arise when one attempts to directly extend EJPS. First, the color channels are spatially separated into Y, Cb, and Cr regions and may be inverted independently, so the RGB covariance matrix *S* used in (7) cannot be reliably estimated. A naive adaptation that treats a single-channel block as a pseudo-RGB triple (e.g., R=G=B=Y) leads to a rank-deficient covariance matrix and an ill-posed inversion. Second, even if such numerical issues were circumvented, the tripling of the number of blocks and the need to consider multiple geometric and photometric transformations would still cause a prohibitive computational burden when combined with the exhaustive search in Equation (6).

These limitations motivate the development of a new attack framework that (i) reduces the effective search space by focusing on structure-rich luminance blocks and pruning redundant evaluations, and (ii) replaces the original RGB-based MGC with a regularized single-channel compatibility measure that remains stable under the grayscale-based EtC architecture. The proposed TBCC, R-SCEC, and Adaptive Pruning components, detailed in Section 4, are designed precisely to meet these requirements.

## 4. Proposed Scheme

### 4.1. Attack Strategy

**Figure 6:**
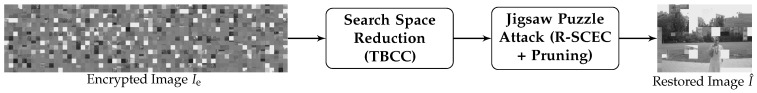
Visual workflow of the proposed attack strategy. The framework simplifies the reconstruction process by first reducing the search space (filtering Cb/Cr blocks) and then performing a specialized jigsaw puzzle attack using the proposed R-SCEC metric with Adaptive Pruning.

The primary objective of this study is to recover visual content from the encrypted image $I_{e}$, thereby demonstrating the security vulnerabilities inherent in the target grayscale-based EtC systems [4,5]. As illustrated in Figure 6, our proposed strategy is formulated by analyzing the failure points of the conventional EJPS [23] when applied to this specific architecture.

Although the EJPS provides a foundation for block assembly, it becomes ineffective against the target system due to structural conflicts introduced by the grayscale serialization. To address these issues, we propose the following specific countermeasures:

- Limitation 1: Computational Explosion (3L blocks).
  →Countermeasure: Dimensionality Reduction via Component Separation (TBCC).
  We exploit the concentration of structural information in the Luminance (*Y*) component. By introducing Texture-Based Component Classification (TBCC), we aim to filter out the majority of Chrominance (Cb, Cr) blocks. This prioritizes assembly of the *Y*-channel, effectively reducing the search space back towards the manageable scale of *L*.
- Limitation 2: Singularity of Covariance Matrix in MGC.
  →Countermeasure: Regularized Single-Channel Edge Compatibility (R-SCEC).
  We propose a mathematical adaptation of the MGC metric by applying Tikhonov regularization to the covariance estimation. By perturbing *S* with a small multiple of the identity matrix, we ensure that the resulting matrix is full-rank and invertible. This allows us to retain the superior texture-matching capabilities of the Mahalanobis distance while operating on single-channel encrypted blocks subjected to rotation, flipping, and luminance inversion.
- Limitation 3: Computational Redundancy.
  →Countermeasure: Adaptive Pruning.
  We implement a mechanism to skip redundant calculations for blocks lacking distinctive features (e.g., uniform edges or mid-gray intensity). By pruning these ambiguous cases, we concentrate computational resources on texture-rich boundaries, thereby enhancing both efficiency and reconstruction accuracy.

The specific implementation details of these components are described in the following subsections.

### 4.2. Search Space Reduction (TBCC)

**Figure 7:**
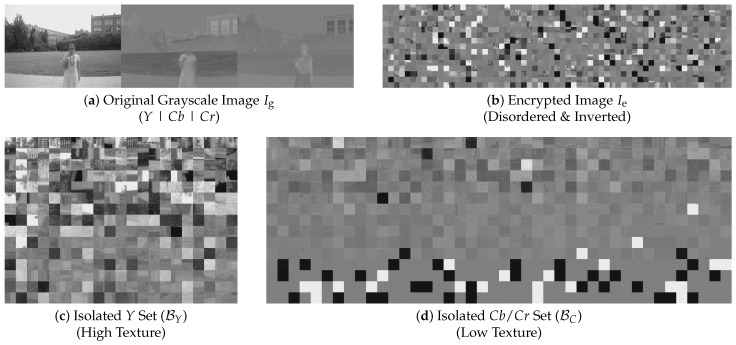
Visualization of the TBCC process. (**a**) Original image (3W) showing *Y*, Cb, Cr concatenation. (**b**) Encrypted image (3W). (**c**) Classified *Y* blocks (approx. 1W) exhibit high texture. (**d**) Classified Cb/Cr blocks (approx. 2W) appear smoother.

The first stage of the proposed framework, as illustrated in Figure 6, aims to drastically reduce the search space dimensionality by isolating the Luminance (*Y*) blocks from the Chrominance (Cb, Cr) blocks. As shown in Figure 7a, the concatenated grayscale image $I_{g}$ exhibits a distinct structural disparity: the left third (*Y*) contains rich high-frequency textures, while the remaining two-thirds (Cb, Cr) are spatially smoother. We leverage this statistical divergence to filter out the low-information chrominance blocks.

#### Feature Extraction and Weighted Fusion

To robustly quantify the information content of each encrypted block $B_{i}$ under the negative-positive inversion ($p\to p^{\prime }=255-p$), we extract a feature vector $f_{i}$ comprising four statistically invariant descriptors.

1. DCT AC Energy ($f_{\mathrm{DCT}}$): We use the sum of absolute DCT AC coefficients; it is invariant to negative-positive inversion up to sign.
2. Gradient Magnitude ($f_{\mathrm{Grad}}$): We use the mean gradient magnitude; inversion flips gradient sign but not its magnitude.
3. Statistical Dispersion ($f_{\mathrm{Std}}$): We use the standard deviation of intensities, which is unchanged under inversion.
4. Information Entropy ($f_{\mathrm{Ent}}$): We use intensity entropy; inversion is bijective and preserves the histogram, hence the entropy.

These features complement each other: DCT captures frequency characteristics, gradient captures edges, standard deviation reflects dispersion, and entropy measures randomness. To integrate them into a single discriminative metric, we define the Texture Complexity Score ($S_{i}$) as a weighted sum of the min-max normalized features $\hat{f}$:

(8) $$S_{i}=0.27\cdot {\hat{f}}_{\mathrm{DCT}}+0.27\cdot {\hat{f}}_{\mathrm{Grad}}+0.25\cdot {\hat{f}}_{\mathrm{Std}}+0.21\cdot {\hat{f}}_{\mathrm{Ent}},$$

where ${\hat{f}}_{k}=\left(f_{k}-\min\left(f_{k}\right)\right)$/$\left(\max\left(f_{k}\right)-\min\left(f_{k}\right)\right)$. The weights were optimized using Fisher Score analysis to maximize the class separability between *Y* and Cb/Cr samples. This relatively balanced distribution reflects the complementary contribution of frequency, spatial, and statistical domains.

Finally, classification is performed by ranking. Exploiting the property of the YCbCr 4:4:4 format where the ratio of *Y*, Cb, and Cr blocks is 1:1:1, we sort all 3L blocks by $S_{i}$ in descending order. The top *L* blocks are classified as the Luminance set $B_{Y}$, while the remaining 2L blocks are categorized as the Chrominance set $B_{C}$. The visual efficacy of this separation is demonstrated in Figure 7.

### 4.3. Jigsaw Puzzle Solver via R-SCEC

Following the search space reduction via TBCC, we obtain a subset of candidate blocks $B_{Y}$ that is expected to predominantly consist of the structure-rich luminance component. The final reconstruction is achieved by assembling these blocks using a modified greedy strategy based on Chuman’s EJPS framework [23]. We introduce two technical enhancements to the conventional solver: an Adaptive Pruning mechanism to reduce computational redundancy, and a Regularized Single-Channel Edge Compatibility (R-SCEC) metric to resolve the mathematical singularity inherent in single-channel gradients.

#### 4.3.1. Adaptive Computational Pruning

Conventional solvers exhaustively compute compatibility scores for all block pairs across all possible transformations. For the target grayscale-based EtC system, the puzzle type corresponds to Type IN (comprising rotation, flipping, and luminance inversion), resulting in a state space of |T|=8×2=16 variations per block.

Although TBCC has successfully reduced the candidate set from 3L to the subset $B_{Y}$ (approximately *L* blocks), exhaustively evaluating all pairwise combinations for the remaining blocks is still computationally intensive and susceptible to mismatches caused by ambiguous textures. To further optimize this process, we introduce a pruning strategy that explicitly targets geometrically and photometrically ambiguous cases:

- Geometric Ambiguity: If a block boundary exhibits low pixel variance (i.e., a nearly flat or uniform region), different geometric transformations (rotation and flipping) yield almost identical boundary profiles. For such edges, the orientation cannot be reliably distinguished, and exhaustive search over all orientations primarily amplifies noise. Therefore, we mark these boundaries as geometrically ambiguous and skip the full orientation search for the corresponding block pairs.
- Photometric Ambiguity: The encryption involves negative-positive inversion ($p\to p^{\prime }=255-p$). For blocks whose mean intensity is close to mid-gray (μ≈128), the statistics before and after inversion become very similar:
  (9) $$\left|p-128\right|\approx \left|\left(255-p\right)-128\right|=\left|127-p\right|.$$
  In this regime, distinguishing the polarity (normal vs. inverted) is unstable and provides little discriminative power. We therefore treat such blocks as photometrically ambiguous and bypass the explicit inversion check when evaluating compatibility.

By skipping transformation states that are unlikely to provide reliable discrimination, Adaptive Pruning concentrates computational resources on texture-rich and informative boundaries. As shown in the experimental section, this not only reduces the overall computation time but also improves reconstruction robustness by mitigating false matches in ambiguous regions.

#### 4.3.2. Regularized Single-Channel Edge Compatibility (R-SCEC)

The core challenge in grayscale EtC cryptanalysis is the breakdown of the MGC metric [16] that underpins the EJPS [23]. The standard MGC relies on the covariance matrix *S* of gradients across R,G,B channels to capture local texture directionality along block boundaries.

In our single-channel scenario, only the luminance component *Y* is available. A naive adaptation that forces a pseudo-color representation (R=G=B=Y) causes the gradient vectors to become linearly dependent. This linear dependence reduces the rank of the 3×3 covariance matrix *S* to one, rendering it rank-deficient (singular). Consequently, the determinant vanishes (|S|≈0), and the inverse matrix $S^{-1}$ required for the Mahalanobis distance is mathematically ill-posed.

To address this ill-posed problem, we introduce Tikhonov regularization. Instead of using the raw covariance, we compute a regularized matrix $S_{\mathrm{reg}}$ by adding a small perturbation to the diagonal:

(10) $$S_{\mathrm{reg}}=S+\epsilon I,$$

where *I* is the identity matrix and $\epsilon =10^{-4}$ is an empirical stabilizer ensuring positive-definiteness under rank-deficient gradients while preserving intrinsic spatial correlations.

Using this stabilized matrix, we define the R-SCEC metric as a single-channel analogue of MGC. For a target block $B_{i}$ and a candidate neighbor $B_{j}$, we consider all possible transformation states *k* (rotation, flipping, and luminance inversion) and compute the dissimilarity along the shared boundary as

(11) $$D_{R-\mathrm{SCEC}}\left(B_{i},B_{j}\right)=\min_{k}\left({\left(g_{ij}^{k}-\mu \right)}^{⊤}S_{\mathrm{reg}}^{-1}\left(g_{ij}^{k}-\mu \right)\right),$$

where $g_{ij}^{k}$ denotes the gradient vector sampled along the boundary between $B_{i}$ and the transformed block $f_{k}\left(B_{j}\right)$, and μ and $S_{\mathrm{reg}}$ are the mean and regularized covariance matrix of these boundary gradients, respectively. Here, μ and $S_{\mathrm{reg}}$ are estimated from the boundary gradient samples of the target block $B_{i}$ (for the corresponding side), following the MGC construction [16].

Importantly, the covariance of a dataset is invariant under global sign flips (e.g., Cov(X,Y)=Cov(−X,−Y)). Therefore, the texture statistics captured by $S_{\mathrm{reg}}$ remain valid even if a block has undergone luminance inversion. Combined with the explicit search over transformation states *k* in Equation (11), this property allows R-SCEC to correctly identify the original block orientation and polarity in the presence of negative–positive encryption. Finally, based on the R-SCEC compatibilities and the Adaptive Pruning strategy, the jigsaw puzzle is assembled using the same minimum spanning tree (MST)–based greedy procedure as in the conventional method [16,23]. Trimming and filling steps are then applied to generate the final reconstructed image $\hat{I}$ with the original spatial resolution.

## 5. Experimental Results

To substantiate the effectiveness of the proposed COA, we conducted a series of experiments focusing on three aspects: (i) The accuracy of component classification by TBCC; (ii) The computational efficiency gained through Adaptive Pruning; and (iii) The visual and semantic quality of reconstructed images under the grayscale-based EtC system.

### 5.1. Experimental Setup and Dataset

**Figure 8:**
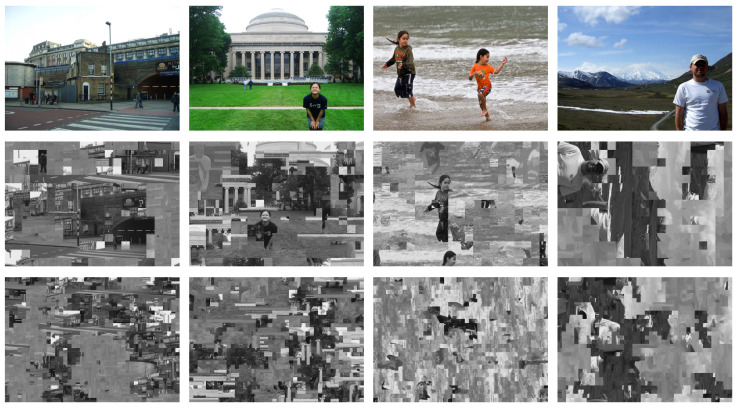
Visual comparison of experimental samples from the MIT Dataset [14]. Top row: Original plaintext images resized to 336×240 pixels. Middle row: Corresponding images reconstructed by the proposed attack from the encrypted state (Type IN) in the Benchmark setting (P=16×16; 3L=945 blocks). Bottom row: Representative reconstruction results in the Canonical setting (P=8×8; 3L=3780 blocks), where reconstruction frequently fails and outputs become visually disordered due to the extreme combinatorial complexity and weaker boundary cues at finer block partitioning.

All experiments were carried out on the MIT dataset [14] (20 images) and the BSDS500 dataset [36] (500 images) with YCbCr 4:4:4 conversion, horizontal concatenation, and block-based visual encryption (permutation, rotation/flipping, and negative–positive inversion, i.e., Type IN in Table 2) [4,5], followed by JPEG compression. Figure 8 shows representative plaintext images (from the MIT dataset) and the corresponding reconstructions obtained with the proposed attack.

The main experimental parameters were determined as follows.

1. Block Size (P=16×16): To ensure a fair and directly comparable baseline against the extended JPS (eJPS) methods that we improve upon, our main setting uses P=16×16, aligning both the block size and the puzzle scale. In line with both the target EtC system [4,5] and typical jigsaw puzzle settings (e.g., 14×14 or 28×28 pixels [23]), we set the block size to P=16×16 pixels. This choice is compatible with JPEG-style block processing while yielding a sufficiently large number of pieces for cryptanalytic evaluation. In addition, to address the canonical pseudo-grayscale setting in Ref. [5], we further evaluate P=8×8 under two complementary configurations described in Item 3 below. If unknown, this parameter can be estimated via statistical periodicity detection or by testing the standard candidates (e.g., 8, 16, 32) constrained by compression efficiency.
2. Chroma Format (Worst-Case Configuration): Although the EtC framework supports chroma subsampling (4:2:0, 4:2:2) [4,5], we deliberately adopted the YCbCr 4:4:4 format. In subsampled formats, luminance (*Y*) blocks dominate in number, making component separation easier. In contrast, 4:4:4 maintains a 1:1:1 block ratio among *Y*, Cb, and Cr, maximizing the proportion of chrominance “distractors”. Demonstrating successful attacks in this hardest case implies applicability to simpler subsampling settings.
3. Image Dimensions and Puzzle Scale: We consider three complementary settings to disentangle the impact of block size and the total number of blocks. All images were resized to 336×240 pixels, ensuring that both dimensions are divisible by the block size (P=16×16 in the main setting, and P=8×8 in the additional evaluations). The YCbCr concatenation yields a grayscale composite of size 3×336×240, which is then partitioned into 3L=945 blocks for the main setting (P=16×16, L=315), and 3L=3780 blocks for the canonical P=8×8 setting at the same resolution (L=1260). To isolate the effect of block size from that of block count, we also report an equal-block-count setting using P=8×8 with a lower resolution of 168×120, which yields the same number of blocks as the main setting (L=315, 3L=945). Compared to the L=432 blocks used in Chuman et al. [23] for RGB-based EtC, our setting produces a significantly larger puzzle, providing a more stringent test of solver robustness, while the two P=8×8 settings allow us to rigorously assess fairness to Ref. [5] and characterize the scalability boundary when the block count increases.

### 5.2. Evaluation of Component Classification (TBCC)

The first stage of the proposed attack is the dimensionality reduction achieved by Texture-Based Component Classification (TBCC). This subsection quantitatively evaluates the accuracy with which TBCC separates luminance blocks ($B_{Y}$) from chrominance blocks ($B_{C}$) in the encrypted grayscale image $I_{e}$.

#### 5.2.1. Test Methodology and Metrics

We consider the worst-case configuration described above, i.e., YCbCr 4:4:4 with the block ratio $B_{Y}:B_{C}=1:2$.

##### Classification Process

For each encrypted image, we compute the texture complexity score $S_{i}$ for all 3L blocks using the weighted fusion model in Equation (8). The blocks are then ranked in descending order of $S_{i}$, and the top *L* blocks are selected as the predicted luminance set $B_{Y}$; the remaining 2L blocks are treated as $B_{C}$.

We use the following three metrics to evaluate classification quality:

- Statistical Accuracy (Acc): The proportion of correctly selected luminance blocks among the top-*L* predictions (i.e., $|B_{Y}\cap B_{Y}^{*}|/L$).
- Benign Error Ratio (BER): Some misclassifications occur in visually homogeneous regions where Y and Cb/Cr are almost indistinguishable. We label an error as benign if the Root Mean Square Error (RMSE) between the misclassified block and its correct-component counterpart is below a perceptual threshold (RMSE<10.0). The BER is defined as the ratio of benign errors to the total number of blocks.
- Effective Accuracy ($Acc_{\mathrm{Eff}}$): Since benign errors have a negligible impact on visual privacy, we treat them as acceptable and define
  (12) $$Acc_{\mathrm{Eff}}=Acc+BER.$$
  This metric is a more realistic indicator of how many blocks are handled correctly for visual reconstruction.

#### 5.2.2. Quantitative Classification Results

**Figure 9:**
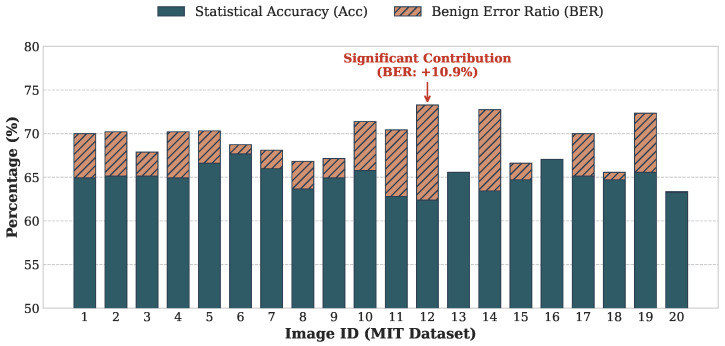
Component classification performance for the MIT dataset [14] under the Benchmark setting (P=16×16; 3L=945 blocks). The blue portions indicate Statistical Accuracy (Acc), and the orange portions indicate the Benign Error Ratio (BER). The total height corresponds to Effective Accuracy ($Acc_{\mathrm{Eff}}$). For images with large smooth regions (e.g., Image 12), benign errors substantially compensate for the drop in raw accuracy.

Figure 9 shows the classification performance for each of the 20 images under the target Type IN encryption (permutation, rotation/flipping, and luminance inversion). The stacked bars visualize the contributions of Acc and BER to the total $Acc_{\mathrm{Eff}}$.

Table 3 summarizes the distribution of these metrics. As presented in Table 3, the proposed TBCC achieves an average Effective Accuracy of approximately 69% in the most challenging YCbCr 4:4:4 configuration. This has two important implications:

- From Blind Search to Guided Attack: In the 4:4:4 format, structure-rich luminance blocks constitute only one third of the data, and random guessing would yield just ≈33.3% accuracy. Achieving ≈69%, therefore, represents roughly a twofold information gain, enabling us to shrink the search space from 3L mixed blocks to a focused set of *L* luminance candidates. This dimensionality reduction is the key that makes the subsequent large-scale jigsaw assembly computationally feasible.
- Semantic Priority over Background Detail: As illustrated in Figure 8, misclassifications primarily occur in homogeneous regions such as sky or grass, where *Y* and Cb/Cr are statistically similar. Recovering precise textures in these areas is much less important than correctly reconstructing semantic foreground objects (e.g., people, buildings). Hence, benign errors do not prevent the attacker from extracting privacy-sensitive visual information.

**Table 3:** Statistical summary of TBCC performance (average, minimum, and maximum) for the 20 images in the MIT dataset [14] under the Benchmark setting (P=16×16; 3L=945 blocks). The results demonstrate a substantial information gain over the random guessing baseline (≈33.3%).

Metric	Average (%)	Minimum (%)	Maximum (%)
Statistical Accuracy (Acc)	64.96	62.38	67.67
Benign Error Ratio (BER)	3.92	0.00	10.90
Effective Accuracy ($Acc_{\mathrm{Eff}}$)	68.88	63.33	73.28

### 5.3. Computational Optimization via Adaptive Pruning

After TBCC, the proposed solver performs pairwise matching on the luminance set $B_{Y}$ using the R-SCEC metric. Under Type IN encryption, a naive brute-force attack needs to test |T|=16 transformation states (4rotations×2flips×2inversionstates) for each block pair, leading to complexity on the order of $O\left(L^{2}\left|T\right|\right)$.

Adaptive Pruning, introduced in Section 4.3.1, mitigates this burden by exploiting two kinds of redundancy: geometric symmetry and photometric ambiguity. Table 4 summarizes the proportion of such cases observed in $B_{Y}$.

**Figure 10:**
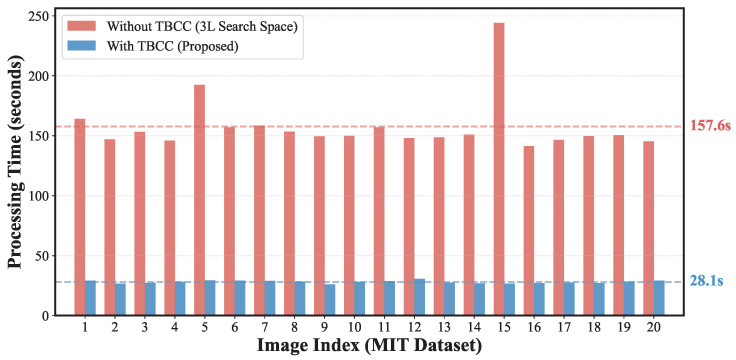
Processing time for the 20 test images from the MIT dataset [14] under the Benchmark setting (P=16×16; 3L=945 blocks). Red bars correspond to the “Blind Search” setting without TBCC; blue bars correspond to the proposed method with TBCC. The results confirm that search space reduction is essential to avoid computational explosion.

Although the fraction of fully symmetric or mid-gray blocks is relatively small in the 336×240 images, they correspond to the most redundant “singular” cases where the state space collapses from 16 to 1. Pruning these states not only reduces the constant factor in the computational complexity but also suppresses spurious matches arising from completely uninformative edges. We further measured the practical impact of TBCC and Adaptive Pruning on runtime. Table 5 shows the average reconstruction time for the 20 images in the MIT dataset [14] under the Benchmark setting (P=16×16; 3L=945 blocks) with and without TBCC (Adaptive Pruning enabled in both cases). The per-image time distribution is illustrated in Figure 10.

Overall, these results confirm that TBCC and Adaptive Pruning together transform a theoretically intractable attack into a practically feasible one, even for puzzles with nearly one thousand encrypted blocks.

### 5.4. Reconstruction Accuracy and Security Boundaries

We evaluate the reconstruction quality achieved by the full proposed framework (TBCC + Adaptive Pruning + R-SCEC) by comparing the reconstructed image $\hat{I}$ with the original luminance component $I_{Y}$. For benchmarking, the baseline EJPS [23] is evaluated against the original concatenated grayscale image $I_{g}$ (3L blocks), while our proposed attack is evaluated specifically against $I_{Y}$ (*L* blocks) to quantify the specific leakage of structural information. As demonstrated in prior research [23], pixel-wise metrics such as SSIM are unsuitable for JPS tasks because minor block misalignments lead to disproportionately low scores despite high semantic intelligibility. Therefore, we adopt the following standard JPS metrics to evaluate reassembly accuracy:

- Jigsaw Puzzle Solver (JPS) Metrics ($D_{c},N_{c},L_{c}$): These represent the standard quantitative measures in the JPS research community [23] for evaluating spatial reassembly accuracy:
  Direct Comparison ($D_{c}$): The ratio of blocks placed at the correct absolute positions:
  (13) $$D_{c}\left(\hat{I}\right)=\frac{1}{n}\sum_{i=1}^{n}d_{c}\left(i\right),\;d_{c}\left(i\right)=\left\{\begin{array}{cc} 1, & \mathrm{if}\;\mathrm{block}\;i\;\mathrm{is}\;\mathrm{at}\;\mathrm{the}\;\mathrm{correct}\;\mathrm{position}\;\mathrm{in}\;\hat{I},\\ 0, & \mathrm{otherwise}. \end{array}\right.$$
  Neighbor Comparison ($N_{c}$): The ratio of correctly joined adjacent block pairs (boundaries):
  (14) $$N_{c}\left(\hat{I}\right)=\frac{1}{B}\sum_{k=1}^{B}n_{c}\left(k\right),\;n_{c}\left(k\right)=\left\{\begin{array}{cc} 1, & \mathrm{if}\;\mathrm{boundary}\;b_{k}\;\mathrm{is}\;\mathrm{joined}\;\mathrm{correctly}\;\mathrm{in}\;\hat{I},\\ 0, & \mathrm{otherwise}, \end{array}\right.$$
  where B=2uv−u−v is the total number of boundaries for a u×v grid.
  Largest Component ($L_{c}$): The size of the largest correctly assembled region normalized by *n*:
  (15) $$L_{c}\left(\hat{I}\right)=\frac{1}{n}\max_{j\in \{1,2,\ldots ,m\}}\left\{l_{c}\left(\hat{I},j\right)\right\}.$$
- SIFT Feature Matching: To quantify the recovery of semantic content, we extract Scale-Invariant Feature Transform (SIFT) features from $\hat{I}$ and $I_{Y}$ and count the number of valid feature matches. We retain this metric for two methodological reasons:
  - Robustness to TBCC Noise: Since TBCC’s effective accuracy is ≈69%, reassembled images inevitably contain chrominance-block “noise” that penalizes reassembly metrics ($D_{c},N_{c},L_{c}$) even if key objects are recovered; SIFT remains robust to such local noise.
  - Semantic Weighting vs. Background Redundancy: Standard metrics treat all blocks equally; thus, reconstructing non-sensitive regions (e.g., sky or grass) can inflate scores without reflecting a privacy breach. In contrast, SIFT keypoints concentrate on feature-rich semantic objects (e.g., people or buildings), providing a more focused assessment of the actual visual privacy leakage risk.

#### Quantitative Analysis and Discussion

**Figure 11:**
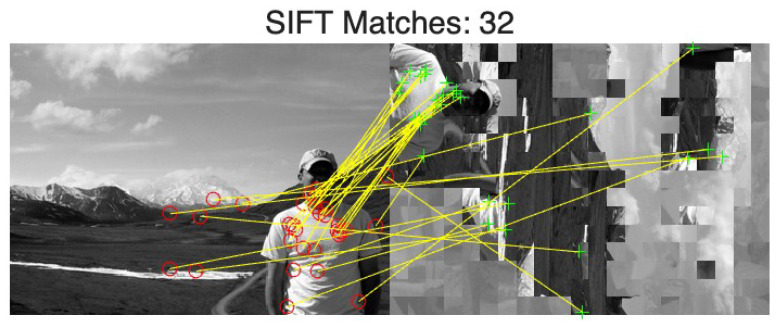
Visualization of SIFT feature matching between the original luminance image $I_{Y}$ (**left**) and the reconstructed image $\hat{I}$ (**right**). The correspondences confirm that critical semantic features remain identifiable despite the absence of color information and block artifacts.

**Figure 12:**
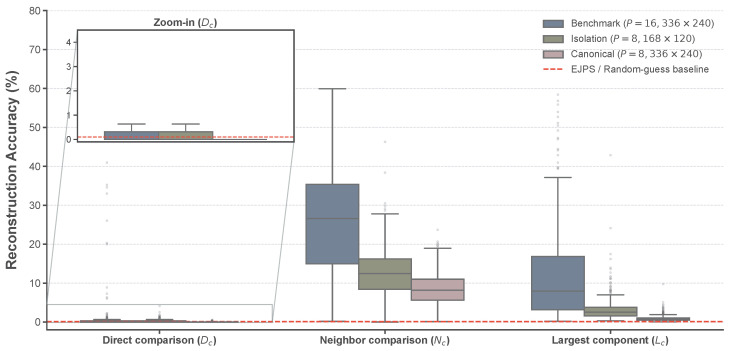
Statistical distribution of $D_{c},N_{c},$ and $L_{c}$ on the BSDS500 dataset (N=500). The red dashed line denotes the EJPS [23] and random-guess baseline (≈0.1%). The results confirm that the proposed attack significantly exceeds the baseline across all configurations.

Figure 11 visualizes the SIFT matches for Image 19, where correspondences are established across primary structural points. Figure 12 presents the distribution of reassembly performance for our method across the BSDS500 dataset [36]. We focus on the distribution of the proposed method, as the EJPS baseline consistently yields near-zero values (≈0.1%) across all metrics, providing no informative variance.

The analysis of Table 6 reveals the critical security boundaries of grayscale-based EtC under different block sizes and puzzle scales. First, EJPS is fundamentally ineffective against this architecture: across all configurations, its reassembly metrics remain at near-random levels ($D_{c}\approx 0.10\%$, $N_{c}\approx 0.10\%$, $L_{c}\leq 0.60\%$) and it produces no valid SIFT correspondences, primarily due to the absence of component separation.

In contrast, the proposed method exhibits clear semantic leakage in the Benchmark setting (P=16×16 at 336×240 pixels), achieving $N_{c}=25.61\;\%$ and $L_{c}=12.99\;\%$, together with an average of 41.8 SIFT matches and a 72.4% success rate for SIFT ≥10. These results indicate that even though the absolute placement accuracy is low ($D_{c}=2.19\;\%$), a substantial fraction of local adjacency relationships is recovered. As highlighted in the magnified inset of Figure 12, the statistical variance of $D_{c}$ in this setting is clearly distinguishable from the EJPS and random-guess baseline. This baseline convergence for EJPS is theoretically expected, as the rank-deficiency of its single-channel covariance matrix renders the Mahalanobis-based matching ill-posed, effectively reducing its performance to a blind search. The proposed attack, by contrast, overcomes this limitation to support reliable object-level correspondence.

When the block size is reduced to 8×8 while keeping the block count comparable (Isolation setting, P=8×8 at 168×120 pixels), performance decreases but remains non-negligible ($N_{c}=12.30\%$, $L_{c}=3.19\%$, 3.2 SIFT matches on average; SIFT ≥10 ratio 14.6%). Given that more than 10 reliable matches are generally considered sufficient to assert semantic correspondence in computer vision applications [37], this result suggests that semantic leakage can still occur at 8×8 under moderate puzzle scales, although it becomes less stable.

**Figure 13:**
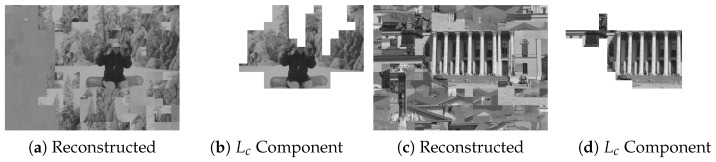
Visual evidence of semantic leakage risk across experimental samples from the MIT Dataset [14] under the Benchmark setting (Type IN, P=16×16, 3L=945 blocks). (**a**,**c**) display the final reconstructed results produced by the proposed attack, while (**b**,**d**) visualize the largest assembled connected component (LCC) that forms the basis for the $L_{c}$ metric. Even under imperfect global reassembly, the LCC effectively isolates high-level semantic structures (e.g., facial features and architectural façades), demonstrating a tangible breach of visual privacy.

To further illustrate the practical implication of such semantic leakage, Figure 13 provides representative qualitative examples from the MIT dataset. As shown in Figure 13a,c, the reconstructed images may contain noticeable global artifacts; however, the corresponding $L_{c}$ component maps in Figure 13b,d visualize the largest assembled connected component (LCC) underlying the $L_{c}$ metric. In these cases, the LCC effectively captures privacy-sensitive semantic structures (e.g., facial features and architectural façades) as a single contiguous region. This confirms that structure-aware attacks can compromise semantic privacy even without achieving perfect aesthetic restoration.

Finally, in the Full-Scale Canonical setting (P=8×8 at 336×240 pixels), the proposed method degrades sharply ($N_{c}=8.31\%$, $L_{c}=0.92\%$). This precipitous drop is mainly attributable to the extreme combinatorial complexity of reassembling 3780 blocks—representing a factorial-scale search space—rather than an intrinsic strengthening of the underlying encryption transform. In summary, grayscale-based EtC is vulnerable to semantic leakage under medium piece counts or larger block sizes, whereas its perceived robustness at very fine partitioning relies on computational complexity rather than inherent algorithmic security.

### 5.5. Ablation Study

To clarify the contribution of each proposed component, we conducted an ablation study by selectively disabling TBCC or replacing R-SCEC with the conventional MGC metric.

#### 5.5.1. Impact of TBCC: Computational Load and Noise

We first removed TBCC and attempted to attack the grayscale-based EtC images using the full set of 3L encrypted blocks, i.e., treating *Y* and Cb/Cr blocks indistinguishably.

##### Computational Load

Without TBCC, the number of candidate blocks increases from *L* to 3L, causing the pairwise matching cost to grow by roughly a factor of nine in theory. The measured processing times in Table 5 (and Figure 10) show an average increase from 28.05 s (with TBCC) to 157.62 s (without TBCC), i.e., a practical slowdown of about 5.6 times. This shows that the blind setting quickly becomes impractical as image size grows.

##### Visual Degradation

**Figure 14:**
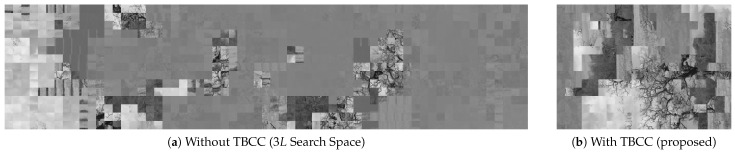
Ablation of TBCC. (**a**) Without component separation, chrominance blocks act as strong distractors and the reconstruction collapses. (**b**) With TBCC, the solver focuses on structure-rich luminance blocks and recovers a coherent image.

In addition, the lack of component separation severely degrades reconstruction quality. Figure 14 compares the reconstructions with and without TBCC. When chrominance blocks are included in the assembly process, the solver frequently confuses smooth chrominance blocks with smooth luminance blocks, particularly in sky or wall regions. These distractors break global consistency and produce chaotic outputs.

TBCC is therefore essential for feasibility and stable reconstruction.

#### 5.5.2. Necessity of the R-SCEC Metric

We next examine the role of the proposed R-SCEC by replacing it with the conventional MGC metric used in [16,23].

Two scenarios were considered:

- Scenario A: Direct application of the standard MGC-based EJPS to the raw encrypted grayscale image (no TBCC).
- Scenario B: Application of the MGC-based solver to the luminance subset $B_{Y}$ obtained by TBCC, giving the conventional metric the advantage of a reduced search space.

**Figure 15:**
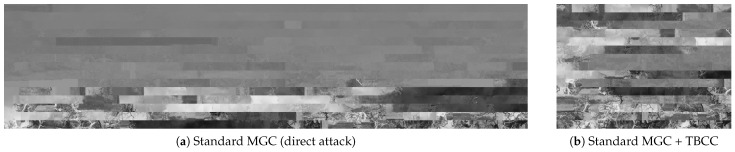
Failure analysis of the conventional MGC-based solver. (**a**) Direct application results in disordered noise due to both metric breakdown and search space explosion. (**b**) Even with TBCC, the single-channel covariance degeneracy makes MGC ineffective for grayscale-based EtC.

In both cases, the MGC-based solver fails to produce meaningful reconstruction, as shown in Figure 15. This double failure highlights two points:

- Search Space Reduction is Not a Panacea: Scenario B shows that even when the correct luminance blocks are isolated by TBCC—providing the conventional metric with an idealized, reduced search space—replacing R-SCEC with the original MGC does not restore attack capability. This failure is particularly noteworthy as it demonstrates that the security of grayscale EtC does not only stem from increased combinatorial complexity, but also from the fundamental degradation of image matching cues (i.e., rank-deficiency in single-channel covariance) which the standard MGC is mathematically unequipped to handle.
- Metric Regularization is Fundamental: As discussed in Section 4.3.2, adding the Tikhonov regularization term in Equation (10) is not a cosmetic modification but a necessary step to make the covariance matrix invertible. By successfully mitigating the rank-deficiency, our R-SCEC restores the discriminative power of edge-based compatibility in the pseudo-grayscale format. Without this regularization, the Mahalanobis distance cannot be computed reliably, and the solver loses its ability to discriminate correct neighbors from incorrect ones.

In summary, the ablation results confirm that both TBCC and R-SCEC are essential: TBCC provides the search space reduction that makes large-scale attacks practical, while R-SCEC restores the discriminative power of edge-based compatibility in the single-channel, luminance-only environment created by grayscale-based EtC encryption.

## 6. Conclusions

This paper has re-examined the security of grayscale-based EtC systems [4,5] against ciphertext-only reconstruction attacks. The central observation is that structural information is unevenly distributed across color channels and that this imbalance is not completely destroyed by YCbCr conversion, horizontal concatenation, and independent block-wise encryption (permutation, rotation/flip, and luminance inversion). To the best of our knowledge, no prior work has successfully mounted a COA on this specific grayscale-based EtC pipeline. Our results, therefore, provide the first concrete evidence that the widely held assumption—that color component separation alone prevents jigsaw-style visual reconstruction—must be critically re-evaluated.

The experimental results support two key findings regarding the feasibility and impact of the proposed attack:

1. Leakage via Component Separation: The proposed TBCC reliably isolates luminance (Y) blocks from the chrominance mixture using only ciphertext statistics. This ability to extract structure-rich components directly from encrypted data reduces the effective search space from 3L mixed blocks to approximately *L* luminance candidates, bringing large-scale jigsaw assembly back into a computationally feasible regime. In addition, Adaptive Pruning operates within the TBCC-reduced search space to skip redundant boundary-matching computations, further improving efficiency while requiring no learning-based training.
2. Semantic Recognizability under Block Artifacts: Although the reconstructed images inevitably contain block artifacts and local misalignments, $D_{C}$/$N_{C}$/$L_{C}$ together with SIFT feature matching shows that a sufficient number of keypoints are preserved to identify semantic content (e.g., faces, buildings, salient objects). In other words, the proposed attack exposes a practical risk of visual information leakage that goes beyond what was anticipated for the target grayscale-based EtC system.

To overcome the mathematical singularity inherent in single-channel gradients, we introduced the R-SCEC metric. By combining Tikhonov-regularized covariance estimation with an Adaptive Pruning mechanism, the solver can robustly handle the joint effects of permutation, rotation/flip, and luminance inversion while avoiding computational explosion. This framework restores the discriminative power of edge-based compatibility in settings where conventional RGB-dependent metrics, such as MGC, fail.

Moreover, experimental results indicate that reconstruction performance degrades as the total number of blocks *L* increases, suggesting an inherent scalability boundary. For ultra-high-resolution images where $L>10^{4}$, the framework faces a computational ceiling where greedy assembly is prone to local optima, making full global reassembly challenging. However, from a security perspective, we assess that even if global reconstruction fails, the risk of local semantic leakage remains a critical threat that grayscale EtC is not designed to mitigate. Further improving attack capability in this regime typically requires rethinking the framework at a more fundamental level, e.g., through improved compatibility modeling or stronger global optimization strategies; from a defensive viewpoint, this also implies that the risk level of grayscale-based EtC can vary with system parameters and scale.

The implications of this study are twofold. First, simply permuting and encrypting separated color channels does not provide robust protection against correlation-based ciphertext-only reconstruction attacks; structural cues concentrated in the luminance component remain exploitable. Second, our findings highlight the need for revised design guidelines for EtC systems, in which security is evaluated not only against brute-force key search but also against structure-aware visual attacks. Based on our observations, a more effective direction is to further suppress boundary cues exploitable by puzzle solvers, e.g., via key-driven mixing/swapping between boundary and interior pixels/regions to reduce the effective information available to edge-compatibility measures and improve robustness against ciphertext-only visual reconstruction.

It should be emphasized that the objective of this work is not to propose a new encryption system, but to provide a security evaluation that reveals previously overlooked vulnerabilities in existing grayscale-based EtC architectures. Future work includes improving the restoration quality (e.g., exploring hybrid frameworks that integrate learning-based refinement) to achieve higher visual fidelity, as well as designing enhanced EtC architectures that explicitly counter luminance-driven reconstruction—for example, through adaptive component mixing, key-dependent chroma processing, or lightweight obfuscation layers that disrupt exploitable correlations while preserving compression compatibility.

## Figures and Tables

**Table 1:** Capability summary of representative jigsaw puzzle solvers, the EtC-oriented Extended Jigsaw Puzzle Solver (EJPS), and the proposed attack in this paper (✓: supported; ✗: not supported).

Method (Ref.)	Rotation	Flipping	Neg.-Pos.	Gray Single-Channel	#Pieces/Piece Size
Cho [14]	✗	✗	✗	✗	432/28×28
Gallagher [16]	✓	✗	✗	✗	9600/28×28
Son [17]	✓	✗	✗	✗	9801/28×28
Sholomon [18]	✓	✗	✗	✗	22,755/28×28
EJPS (Chuman) [23]	✓	✓	✓	✗	432/28×28
Proposed attack (ours)	✓	✓	✓	✓	945/16×16 (main)

**Table 2:** Comparison of search space complexity for different jigsaw puzzle types. The RGB-based EtC system corresponds to Type INC.

Puzzle Type	Active Encryption Operations	Symbol	Variations per Block |T|
Type 1	Permutation only	–	1
Type 2	+Rotation ($90^{\circ }\times 4$)	*R*	4
Type I	+Flipping (H/V)	R+F	4×2=8
Type N	+Neg–Pos inversion	R+N	4×2=8
Type IN	+Rotation, Flipping, Neg–Pos	R+F+N	8×2=16
Type INC	+Color Component Shuffling	R+F+N+C	8×2×6=96

**Table 4:** Analysis of computational redundancy within the Luminance Set $B_{Y}$. The “Complexity Reduction” describes how the effective state space size |T| collapses for specific block types.

Redundancy Type	Condition	Complexity Reduction	Ratio (%)
Geometric (Rot/Flip)	2 edges symmetric	|T|×0.5	0.78%
	3 edges symmetric	|T|×0.25	1.40%
	4 edges symmetric	8→1 (Geom. States)	2.22%
Photometric	Edge pixels ≈127.5	2→1 (Inv. States)	0.16%
Combined	Fully flat & mid-gray	|T|:16→1	<0.1%

**Table 5:** Comparison of computational time (seconds) with and without TBCC for the MIT dataset [14] under the Benchmark setting (P=16×16; 3L=945 blocks). Values are averaged over 20 images (336×240 pixels). Adaptive Pruning is enabled in both configurations.

Configuration	Search Space	Avg. Time (s)	Std. Dev.	Speedup
Without TBCC	3L (Mixed)	157.62	22.86	1.0× (Baseline)
With TBCC (Proposed)	*L* (Luminance only)	28.05	1.18	5.62×

**Table 6:** Comparative summary of reconstruction accuracy (average) and semantic leakage success rate over the dataset for the BSDS500 dataset [36] (N=500). EJPS is compared against $I_{g}$, while the Proposed method is compared against $I_{Y}$.

Configuration	Method	$D_{c}$ (%)	$N_{c}$ (%)	$L_{c}$ (%)	SIFT Matches	SIFT ≥10 Ratio (%)
Benchmark	EJPS	0.10	0.10	0.60	0.0	0.0
(P=16×16 at 336×240 pixels)	Proposed	2.19	25.61	12.99	41.8	72.4
Isolation	EJPS	0.10	0.10	0.03	0.0	0.0
(P=8×8 at 168×120 pixels)	Proposed	0.16	12.30	3.19	3.2	14.6
Canonical	EJPS	0.10	0.10	0.01	0.0	0.0
(P=8×8 at 336×240 pixels)	Proposed	0.04	8.31	0.92	0.2	1.2

## Data Availability

The data presented in this study are available in the MIT dataset at https://people.csail.mit.edu/taegsang/JigsawPuzzle.html (accessed on 11 December 2025), and in the BSDS500 dataset at https://www2.eecs.berkeley.edu/Research/Projects/CS/vision/grouping/resources.html (accessed on 27 January 2026).
